# Long-lasting upper ocean temperature responses induced by intense typhoons in mid-latitude

**DOI:** 10.1038/s41598-022-09833-2

**Published:** 2022-04-06

**Authors:** Jun-Hyeok Son, Ki-Young Heo, Jung-Woon Choi, Jae-il Kwon

**Affiliations:** Korea Institute of Ocean Science and Technology, Busan, Korea

**Keywords:** Atmospheric science, Climate sciences, Ocean sciences

## Abstract

The sea surface temperature (SST) drops rapidly when a typhoon passes over the western North Pacific, and the cold SST is known as cold wake. In general, more intense typhoons on the day of arrival cause stronger SST cooling via turbulent oceanic vertical mixing. Moreover, after intense typhoons have passed, there are cases in which the SST decreases further, and the cold conditions persist for approximately 2 weeks. In this study, we suggest possible mechanisms by which long-lasting cold SST responses to typhoon forcing are related to the generation of cold-core-like ocean circulation. The atmospheric surface cyclonic circulation causes divergent anticlockwise upper ocean currents owing to the Ekman transport, which in turn induces further upwelling and strengthens the cold SST. In the European Center for Medium-Range Weather Forecasts Ocean Reanalysis System 5, cold-core-like ocean current responses were strong in 5 typhoons among the 12 intense typhoons that passed through 30°N in the western North Pacific region from 2001 to 2019. The favorable conditions for a cold-core circulation to occur can be summarized as a slow typhoon migration speed with strong intensity, well stratification of vertical ocean layers, and the absence of large-scale strong background currents.

## Introduction

Typhoons are important weather phenomena that have a significant impact on the environment and human activities over the northwest Pacific and East Asia^1^. Typhoons are usually accompanied by heavy rainfall, strong winds and low pressure, causing storm surges, swell waves, wind-driven Ekman currents, and vertical ocean mixing^2–8^. The ocean vertical mixing or upwelling generally causes cold temperature and additional biogeochemical processes such as increased nutrient supply, primary production, and particulate organic carbon flux^9–18^. Cold sea surface temperature (SST) anomalies known as “cold wakes” occur following the typhoon trajectory^19–22^. When a strong typhoon impacts on the ocean, the SST drops by approximately to 8 °C, as per in situ observations, and cold wakes continue for days to weeks^2,23^. The occurrence of a cold wake has a significant impact on the marine environment and ecosystem; in the case of a slow-moving typhoon, the cold wake interacts with the typhoon and plays a role in reducing the typhoon precipitation by ~ 15%^24^.

Cold wakes occur following the typhoon track, and the major SST cooling mechanisms are known as follows: (1) radiative cooling induced by the reflection of solar short-wave radiation^25^, (2) wind-driven enhanced evaporation^26^; (3) oceanic turbulent vertical mixing^27^, and (4) upwelling by Ekman transport^28^. Except for radiative cooling, all other processes are related to surface wind forcing. Many prior studies have shown that stronger typhoon surface winds lead to stronger ocean upwelling, turbulent mixing, and cold temperature responses^29,30^. Typhoon strength can be defined as the maximum wind speed or minimum pressure. In Table S1, the typhoon intensity category sorted by 1 min mean maximum wind speed based on the Saffir–Simpson scale, which is the USA standard, is shown.

**Figure 1:**
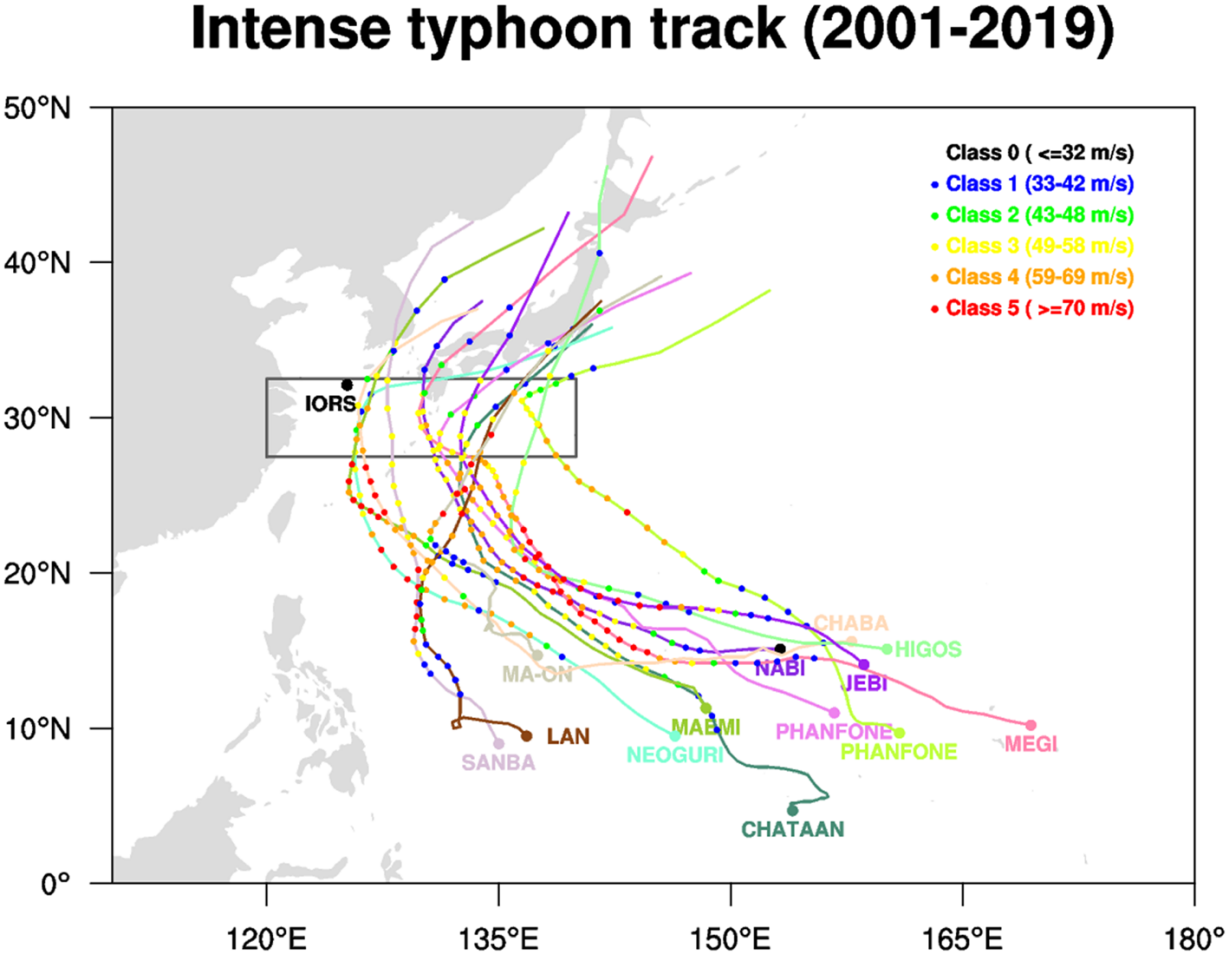
Typhoons with intensities above class three coming through the domain [120°–140°E, 27.5°–32.5°N] from 2001 to 2019 (the map was drawn using NCAR Command Language Version 6.4.0; https://www.ncl.ucar.edu).

Wind-driven upper ocean vertical mixing is the primary cause of cold wakes^27^. However, the reasons why a cold SST persists for a few days or weeks after a typhoon has passed have not been clearly elucidated. This study analyses the possible physical mechanisms that maintain the cold SST after an influence of vigorous typhoon. However, in-situ observations are rare for a strong typhoon to pass directly through, and even when we have that, the observed data is often not available properly due to instrument failure. Therefore, the ocean dynamical and thermodynamical response induced by strong typhoon forcing was analyzed using up-to-date ocean reanalysis and satellite observation data, for typhoons passing through the western North Pacific domain [120°–140°E, 27.5°–32.5°N] with intensity classes above 3 (Fig. 1 and Table S2). However, sometimes the satellite and reanalysis data do not represent well the real ocean conditions. Therefore, we have performed the comparison of the datasets with in-situ observations in the Ieodo station. The Ieodo Ocean Research Station (IORS) is located 149 km southwest of Jeju Island in Korea (125.18°E, 32.12°N), in the path of many typhoons in the East China Sea. It was established in June 2003^31,32^; however, there is no intense typhoon passing over the IORS. Meanwhile, three typhoons (intensity category 0–1) passed near the IORS in 2019; Typhoon Danas (16 July, 2019–20 July, 2019) was the closest to the IORS on 20 July, 2019, Lekima (04 August, 2019–12 August 2019) was closest to the IORS on 11 August, 2019, and Lingling (02 September, 2019–08 September, 2019) was on 07 September, 2019. A rapid SST drop was observed at the IORS, and even in the OISST satellite data when these typhoons passed through the adjacent ocean; the cold SST persisted for 1–2 days before returning to normal state (Fig. S1). The SST variations in 2019 do not represent the SST response to the strong typhoon; however, SST cooling via vertical mixing was clearly observed when the typhoons approached the IORS (Figs. S2 and S3).

## Characteristics of typhoon and sea surface temperature response

**Figure 2:**
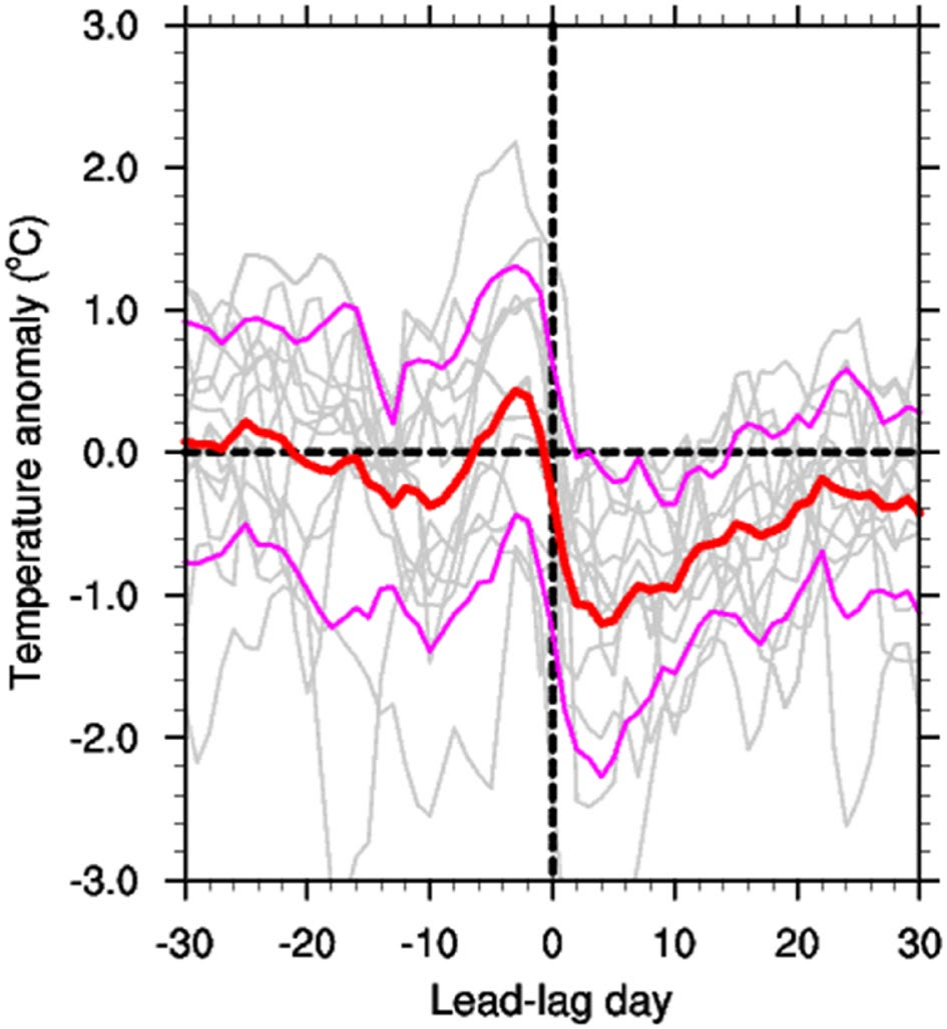
Lead-lag temperature anomaly when typhoons were located at 30°N. The red line shows the average of 12 typhoons, the magenta lines denote ± 1 standard deviation, and gray lines represent individual typhoons. Day 0 denotes typhoon arrival day.

The steering wind and beta drift are the primary determinants of typhoon tracks^33–35^. In general, as seen in Fig. 1, typhoons formed in the tropical western Pacific moves northwestward due to beta drift, then re-curve northeastward when influenced by the western North Pacific subtropical high or the so-called steering wind. In other words, over the tropical western Pacific, typhoons propagate northwestward owing to internal dynamics, and the external steering wind then pushes them northeastward toward Korea and Japan. The typhoons gradually strengthen until they reach the re-curving point (~ 30°N), and the maximum intensity occurs between 10°–25°N. As shown in Fig. 1, except for Chataan, every typhoon reached the strongest category 5, after which they moved further north to 30°N and gradually weakened, falling in categories 2–4. Before the typhoon arrival day, the SST at 30°N was the same or slightly warmer than the normal conditions owing to the solar short-wave radiation. However, as shown in Fig. 2, the SST dropped sharply on the day of typhoon arrival compared to the normal, and the minimum SST was noted approximately four days after the typhoon passed away. On average, the cold SST conditions prevailed for approximately 2 weeks.

The ocean subsurface temperature 5 days before the typhoon arrival can be regarded as the normal state or climatology (Fig. S4). The mixed layer is generally deep in the tropics, but shallower in mid-latitudes during boreal summer^36^. This implies that the normal state mixed layer is shallower in the mature or decaying phases, while that is deeper in the developing early stages of the typhoon. In here, developing (decaying) phase of the typhoon means strengthening (weakening) period of the typhoon intensity in tropics (mid-latitudes), and the mature phase denotes the strongest period in subtropics. These background thermodynamic characteristics are shown in Fig. S4, for each typhoon case. The temperature anomaly 5 days after the typhoon arrival is shown in Fig. S5. The overall subsurface temperature anomaly along the typhoon track 5 days after its arrival was negative. The ocean response to the typhoons showed no consistency (Fig. S5) during their initial phase, from 0 to + 3 days, as the wind forcing was not that strong owing to the weak typhoon intensity, and the ocean temperature and currents were under the influence of large-scale tropical atmospheric and oceanic phenomena, such as the Madden or Julian Oscillation and El Niño Southern Oscillation. During the mature period of a typhoon, the oceanic cold temperature response is the strongest owing to the intense wind speed. The timing of the strongest mature phase is not the same for all typhoons because the duration of individual typhoons is not the same due to different background oceanic and atmospheric conditions. During the decaying phase of typhoon, the last 2–3 days, the ocean response varies again because of weak typhoon forcing. The vertical structure of the ocean subsurface temperature condition is another cause for the disparity in the oceanic temperature response throughout a typhoon’s lifespan. During the developing (decaying) phase of typhoons, the climatological thermocline depth is usually deep (shallow) over the tropics (high latitudes) as shown in Fig. S4^36^; however, the background temperature distribution is not the always same before the typhoon events due to influence of various climate factors.

**Figure 3:**
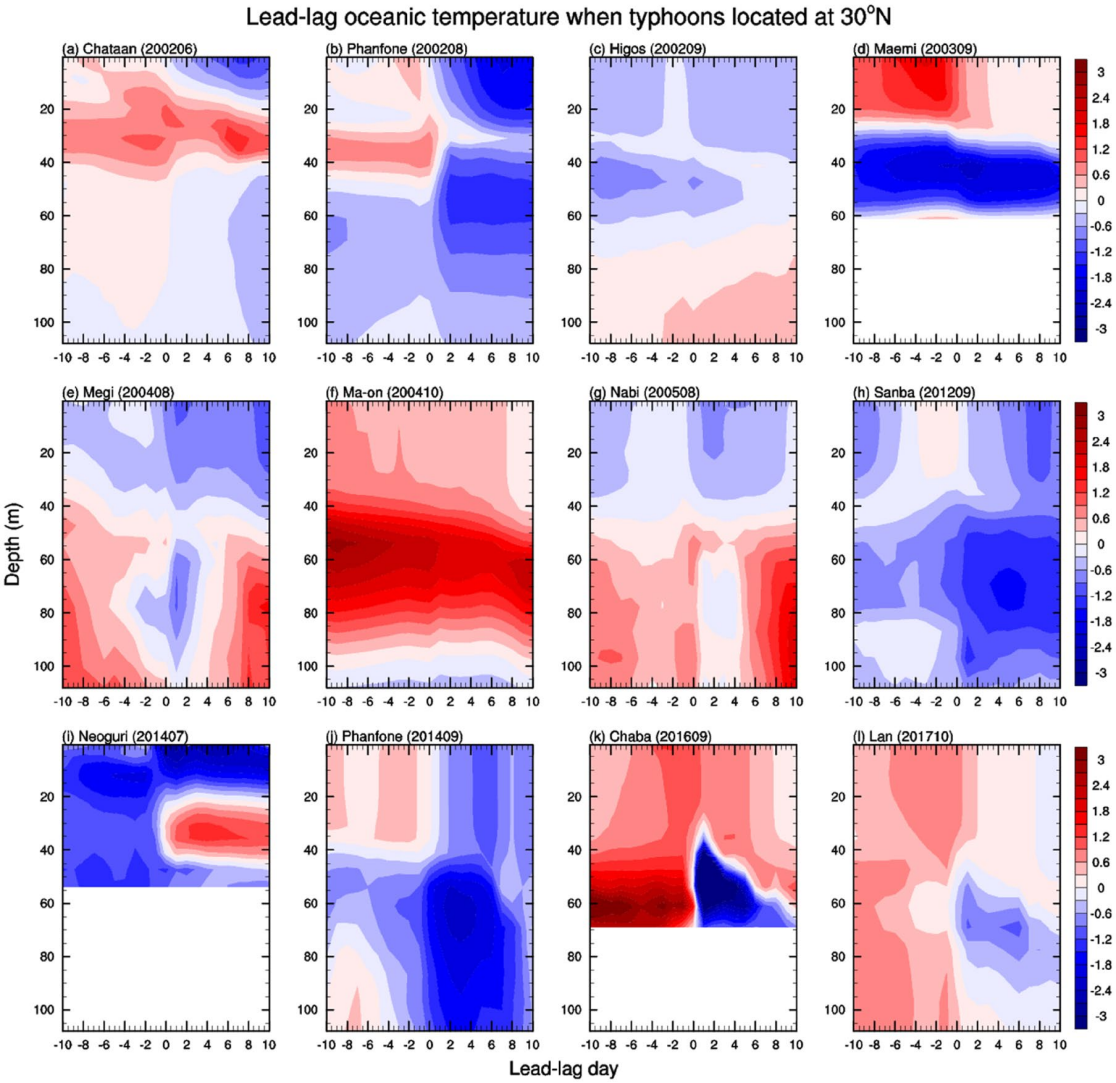
Lead-lag vertical profiles of the temperature anomaly with respect to typhoon arrival day. When typhoons were located at 30°N, the vertical profiles were plotted from -10 to 10 days for 12 individual typhoon cases.

Individual typhoon moves with different speeds; thus, the duration for which a typhoon affects the ocean at a certain location is not the same, and a slow typhoon propagation speed is favorable for ocean- and thermo-dynamic responses^37–39^. Not only does each typhoon’s mean migration pace differ, but each typhoon’s propagation speed also varies during its lifecycle (Fig. S6). Typhoons slowly move to the north in the tropics, but they gradually accelerate moving northward. Around 30°N, the propagation speed of Higos, Ma-on, and Lan were too fast to affect the ocean conditions. See the method section for the typhoon migration speed and favorable ocean condition to generate the cold wake. Except for these three cases, all typhoons caused significant cooling in the upper ocean (to 30 m) on day 0, and cold temperature anomalies persisted after the typhoon passed, as seen in Fig. 3; here, zero-day refers to the arrival of a typhoon; negative day refers to the days before, and positive day is after the typhoon. In the case of Maemi, a strong negative temperature tendency occurred, but the temperature did not reach to the negative anomaly like the other case because the preceding ocean temperature was too warm. In case of Chaba, another exceptional ocean response was noted. There was no cold water under the mixed layer because of the shallow ocean depth.

## Possible dynamical mechanism for the long-lasting cold SST response

After the strong typhoon passed away, the cold temperature anomalies are continued for a few days in the upper ocean layer. The interesting fact is that the cold temperature peak appeared approximately 4 days after the typhoon passed away, as shown in Fig. 2. A possible mechanism for this is the formation of a cold-core-like eddy, wherein the anticlockwise ocean current is driven by typhoon cyclonic wind forcing. If only turbulent mixing induces a cold SST, the temperature may recover shortly; however, a cold-core-like current can maintain a negative SST anomaly for a longer period via oceanic upwelling. In the T-S diagram (Fig. S7), the ocean vertical mixing or upwelling from subsurface to surface can be inferred since the typhoon affecting day in overall cases. In Fig. S7, the superposition or nearness of surface and subsurface dots denote the ocean vertical mixing. However, in case of Neoguri, there is big spread in salinity at the surface layer, and this may be influenced by rainfall or freshwater infusion. The ocean current vorticity and divergence are shown in Figs. S8 and S9, where the blue and red shading shows the counterclockwise and clockwise currents along with divergence and convergence, respectively. The cyclonic wind forcing of Phanfone02, Maemi, Megi, Phanfone14, and Chaba induced long-lasting anti-clockwise ocean currents for 4–8 days; however, Chataan, Nabi, Sanba, and Neoguri caused short-lived cold-core-like currents for 1–2 days (Fig. S8). The upper layer divergence is generated by anticlockwise rotation (Fig. S9), which can result in oceanic upwelling and SST cooling. With the exception of Maemi, which may be related to nonlinear vertical pumping, every other strong typhoon produced an upper-level divergent current^40^. Divergent currents were the strongest on typhoon arrival day owing to direct typhoon wind forcing, while those occurred intermittently afterwards because of the sustained cold-core-like current.

**Figure 4:**
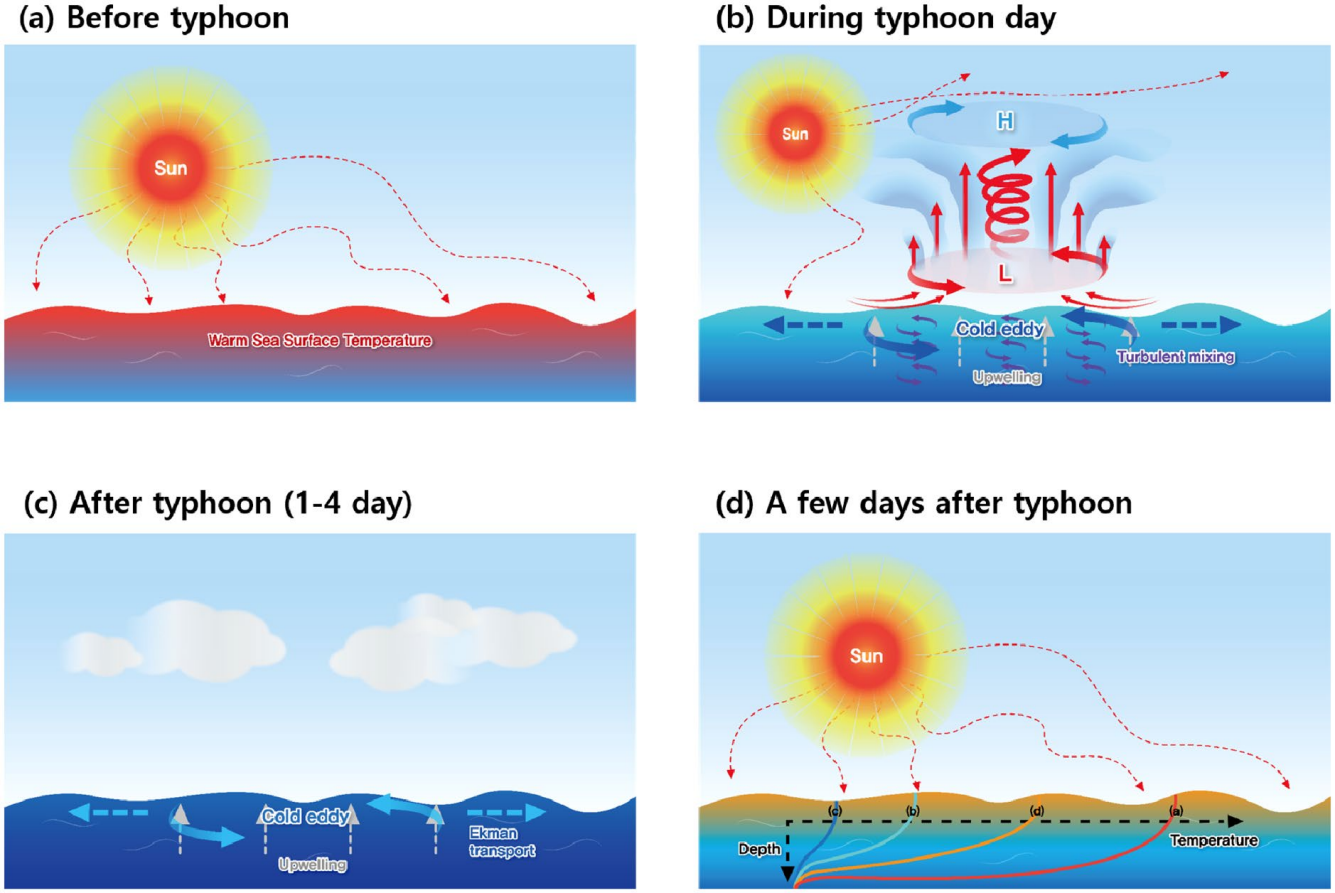
Schematics of physical processes of ocean temperature change before typhoon (**a**), during typhoon affecting day (**b**), after typhoon (**c**), and a few days after typhoon arrival (**d**). Vertical temperature profiles in each time step are shown in (**d**).

In Fig. 4, we have summarized the physical mechanisms responsible for ocean responses to typhoon forcing. Before the typhoon’s arrival, the ocean layer was vertically well stratified, which means that the upper layer was warm, and the temperature gradually decreased in deeper water (Fig. 4a). Owing to solar radiation, warm SST anomalies tend to exist before typhoons (Fig. 2). However, the prior SST conditions can be diverse because of multiple reasons, such as when another typhoon passes on a similar path right before, a strong temperature advection induced by a large-scale current, and a solar insolation changes owing to weather and climate phenomena.

On typhoon arrival day, rapid SST cooling occurs due to the reflection of solar radiation, enhanced evaporation driven by strong surface wind forcing, turbulent ocean vertical mixing, and upwelling by Ekman transport (Fig. 4b). A strong negative SST tendency on the typhoon arrival day induced by vertical mixing or upwelling was shown in the IORS observations, OISST satellite, and ORAS5 datasets (Figs. 2, 3; Figs. S1, S2, S3 and S7). The SST decreased even further after the powerful typhoons passed, and the SST minimum occurred on the fourth day on average (Fig. 2); in which the temperature response is related to the oceanic dynamical process as seen in Figs. S5, S6, and Fig. 4c. The typhoon-induced cold SST anomalies lasted for 2 weeks returning to normal state in average (Fig. 2). Radiation, surface heat flux, and large-scale ocean heat transfer all together play a role in restoring processes to normal conditions^41^.

## Summary and discussion

Of the 12 strong typhoons that passed through the northwest Pacific from 2001 to 2019, 5 typhoons generated cold-core-like horizontal currents at 30°N, wherein the anticlockwise vortex current and divergence were induced, and the cold SST lasted for 2 weeks. In particular, the cold SST peak was noted 4 days after the typhoon passed away. Favorable conditions and possible mechanisms for future SST cooling and long-lasting cold SST signals are as follows:

1. Strong typhoon wind forcing at the time (intensity category 2–4).
2. Slow typhoon migration speed for the generation of ocean currents.
3. Ocean layer vertical stratification for upwelling of subsurface cold water.
4. Mid-latitude location with large planetary vorticity to generate cold-core-like vortex currents by the Ekman transport.

The rapid SST cooling on typhoon affecting day is definitely caused by turbulent vertical mixing; however, the delayed negative peak and cold SST over a long period may be related to the anticlockwise ocean current and the subsequent upwelling. The cold SST persists longer in the subtropics or at mid-latitudes than that in the tropics (not shown) because the Ekman transport and anticlockwise cold-core-like vortex current are stronger at higher latitudes owing to the large planetary vorticity. Meanwhile, the magnitude of the cold SST anomaly peak is stronger when a typhoon is located south of 30°N due to the stronger typhoon forcing.

Different background conditions such as large-scale currents and mixed layer depths can cause diverse ocean responses. For example, the ocean circulation and vertical temperature structure in the open ocean and near shore are different. In addition, as shown in Fig. S10, the typhoon’s wind shape can induce different responses in ocean currents with Ekman transport. According to the surface wind direction, the wind-driven Ekman current and cold water upwelling responses can differ. After the rapid SST cooling induced by turbulent mixing, if the current in that area has a structure of anticlockwise cold-core-like vortex current induced by typhoon wind forcing, a cold SST may be sustained for a long period. However, if only upper-level ocean divergence occurs, rather than a formation of vortex flow, the local SST may quickly return to the recovery phase. Depending on the structure of the typhoon, its radius, and the distance from the center, the portion of the divergent and rotational wind components can be changed. To elucidate this series of physical processes, further in-depth research using a high-resolution atmosphere–ocean coupled dynamical model. In particular, the use and validation in abundant in-situ observations is needed to reduce uncertainties.

## Data and methods

The Joint Typhoon Warning Center of the U.S. Naval Pacific Meteorology Oceanography Center in Hawaii (in Guam, before 1999) best-track datasets from 2001 to 2019 were extracted (https://www.metoc.navy.mil/jtwc/jtwc.html?western-pacific). The best track data are available from 1945; however, detailed typhoon structure and minimum pressure observation data are available from 2001. From 2001 to 2019, 13 typhoons with intensity category three typhoons coming through the western North Pacific domain (120°–140°E, 27.5°–32.5°N) were listed in Table S2; however, typhoon Jebi was excluded from this analysis because the previous typhoon Cimaron migrated in almost the same path, disrupting ocean temperature and circulation. There were two typhoons named Phanfone in 2002 and 2014; we refer to them as Phanfone02 and Phanfone14, respectively. For all typhoons listed in Table S2, a maximum radius (km) of 49 knots was downloaded from the Korea Meteorological Administration website (https://www.weather.go.kr/w/typhoon/typ-history.do).

The daily product of the European Centre for Medium-Range Weather Forecasts Ocean Reanalysis System 5 (ORAS5)^42,43^ daily product is available (https://www.ecmwf.int/en/forecasts/dataset/ocean-reanalysis-system-5) from 1993 to 2019, currently; however, we downloaded data from 2001 to 2019 corresponding to the period of other dataset. The anomaly values shown in this study is calculated as the deviation from the daily climatological calendar mean from 2001 to 2019. The ORAS5 data is made by assimilating the satellite and global in-situ observations^44^, and has an eddy-permitting 0.25° horizontal resolution and 75 vertical layers from 0.5 to 5902 m. In this study, the upper 25 layers from 0.5 to 108 m were analyzed to focus on the wind-driven currents and upper-level thermodynamic structures. Horizontal divergence and vorticity were calculated using the centered finite difference method from the zonal and meridional components of the currents. Daily NOAA Optimum Interpolation Sea Surface Temperature version 2 (OISST)^45^ satellite data (https://psl.noaa.gov/data/gridded/data.noaa.oisst.v2.highres.html) were used for the same period as the other datasets. However, the satellite observations and ocean reanalysis data are not always fit well with the in-situ observations regarded as true values. Therefore, for the validation, we have compared the satellite and ORAS5 data with in-situ observation. In situ measurements at the Ieodo Ocean Research Station (IORS) are provided to the research community via the Korea Ocean Research Stations Project website (http://kors.kiost.ac.kr/en/). Danas, Lekima, and Lingling were typhoons that closely approached or passed through IORS in 2019, but they all had an intensity of 0–1 at approach. Figure S1 shows the OISST (orange line) and IORS (blue line) SST time series for 2019. The detailed characteristics of two timeseries were different, but the SST typhoon responses were similar in each other, so the analysis conducted using OISST data rather than IORS data. In addition to this, the comparison between in-situ observation and ORAS5 reanalysis data were performed as shown in Fig. S3. The T-S diagram shows the vertical mixing in both datasets induced by typhoon forcing. There was much discrepancy in absolute values between two datasets; however, the rough response to the typhoon forcing is similar each other.

## Favorable condition for the generation of cold wake

The phase velocity of the first baroclinic shallow water wave C is defined as $\sqrt {\varepsilon gH_{1} }$^46^. Here, $\varepsilon = (\rho_{2} - \rho_{1} )/\rho_{2}$, *g* = 9.8 m s^−2^, and *H*_1_ = 30 m (based on Fig. S4). The idea is that the migration speed of atmospheric typhoon wind forcing should be slower than the phase speed of oceanic wave to generate the cold wake. Assuming that the density difference between the two vertical layers is large enough ~ 10%, the phase speed C is approximately 59 km h^−1^, and the typhoon migration speed is about 23 ± 15 (1 STD) km h^−1^ at 30°N in the Northern Hemisphere^47^. In the high latitude, the typhoon moving speed tends to become faster; on the contrary, the oceanic baroclinic wave speed is slower due to shallow upper ocean layer depth.

## Supplementary Information

Supplementary Information.

## Data Availability

All datasets analyzed in current study are publicly available.
