# Supplementary material for: Long-lasting upper ocean temperature responses induced by intense typhoons in mid-latitude
Source: Sci Rep. 2022 Apr 6;12:5752. doi: 10.1038/s41598-022-09833-2 (PMC8987045; doi:10.1038/s41598-022-09833-2)
Supplement: Supplementary file 1 — Supplementary Information. [file 41598_2022_9833_MOESM1_ESM.docx]

**Supplementary information**

Table S1. Typhoon intensity category sorted by maximum wind speed using Saffir–Simpson scale.

| Category | Maximum wind speed (knots) | Maximum wind speed (m/s) |
| --- | --- | --- |
| 0 | ≤63 | ≤32 |
| 1 | 64–82 | 33–42 |
| 2 | 83–95 | 43–48 |
| 3 | 96–113 | 49–58 |
| 4 | 114–135 | 59–69 |
| 5 | ≥135 | ≥70 |

Table S2. Typhoons coming through domain [120°–140°E, 27.5°–32.5°N] with an intensity class stronger than 3.

| Name | Period | Minimum pressure (hPa) | Maximum wind speed (knots) | Maximum radius of 49 knots wind (km) |
| --- | --- | --- | --- | --- |
| Chataan | 27/Jun/2002 – 10/Jul/2002 | 910 | 130 | 550 |
| Phanfone | 10/Aug/2002 – 20/Aug/2002 | 904 | 135 | 830 |
| Higos | 25/Sep/2002 – 02/Oct/2002 | 904 | 135 | 650 |
| Maemi | 04/Sep/2003 – 13/Sep/2003 | 885 | 150 | 550 |
| Megi | 17/Aug/2004 – 31/Aug/2004 | 879 | 155 | 520 |
| Ma-on | 01/Oct/2004 – 09/Oct/2004 | 898 | 140 | 400 |
| Nabi | 28/Aug/2005 – 06/Sep/2005 | 898 | 140 | 700 |
| Sanba | 10/Sep/2012 – 18/Sep/2012 | 907 | 155 | 530 |
| Neoguri | 02/Jul/2014 – 11/Jul/2014 | 918 | 140 | 490 |
| Phanfone | 27/Sep/2014 – 06/Oct/2014 | 922 | 135 | 420 |
| Chaba | 25/Sep/2016 – 05/Oct/2016 | 911 | 150 | 380 |
| Lan | 15/Oct/2017 – 23/Oct/2017 | 922 | 135 | 530 |
| Jebi | 27/Aug/2018 – 04/Sep/2018 | 907 | 155 | 340 |


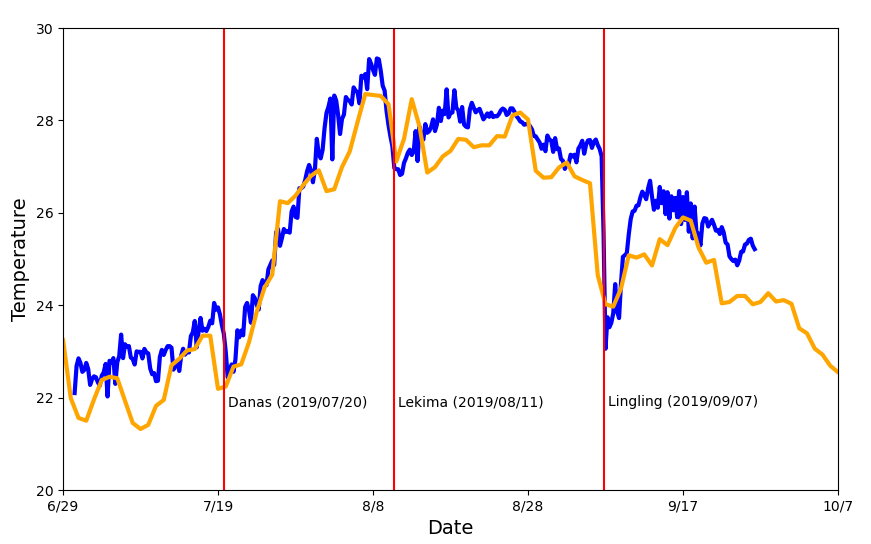


Figure S1. In-situ observation of sea surface temperature at the Ieodo ocean research station (125.18°E, 32.12°N; blue line), and daily NOAA optimum interpolation sea surface temperature (OISST) version 2 satellite data (125.125°E, 32.125°N; orange line) in 2019. The observations at Ieodo are plotted as 6 hourly means, and OISST data are daily means. Red lines show when the typhoons were closest to the Ieodo station.


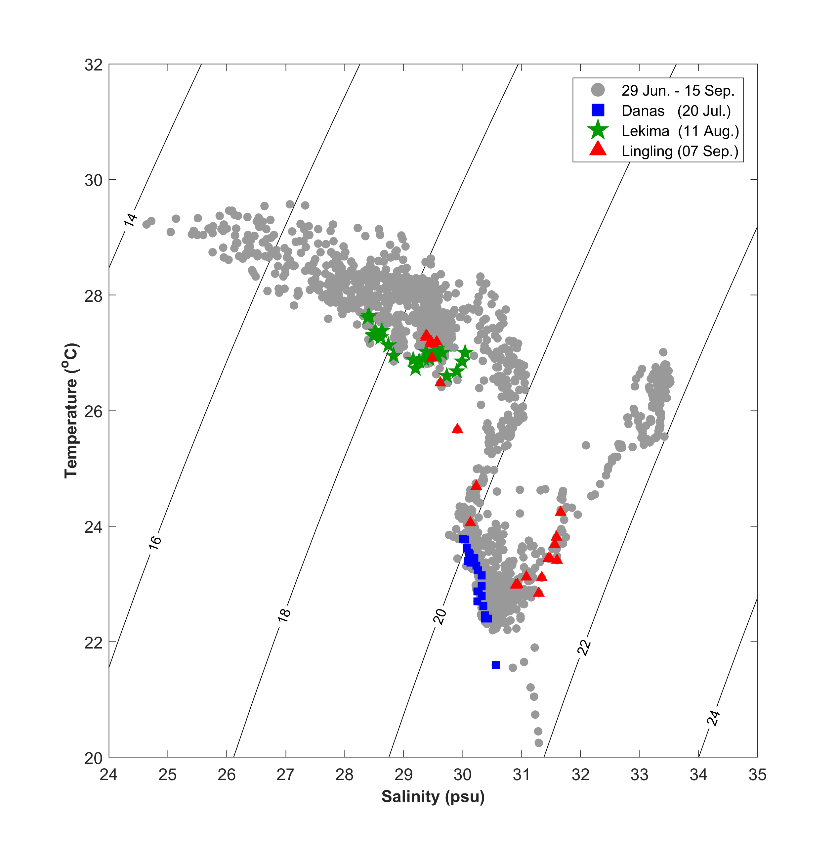


Figure S2. Surface T-S diagram observed with hourly interval in IORS during 29 June – 15 September in 2019. Blue dots show the day affected by Danas (20 July), green is Lekima (11 August), and red is Lingling case (07 September). The observation data is plotted in 24 hours with 1 hour interval.


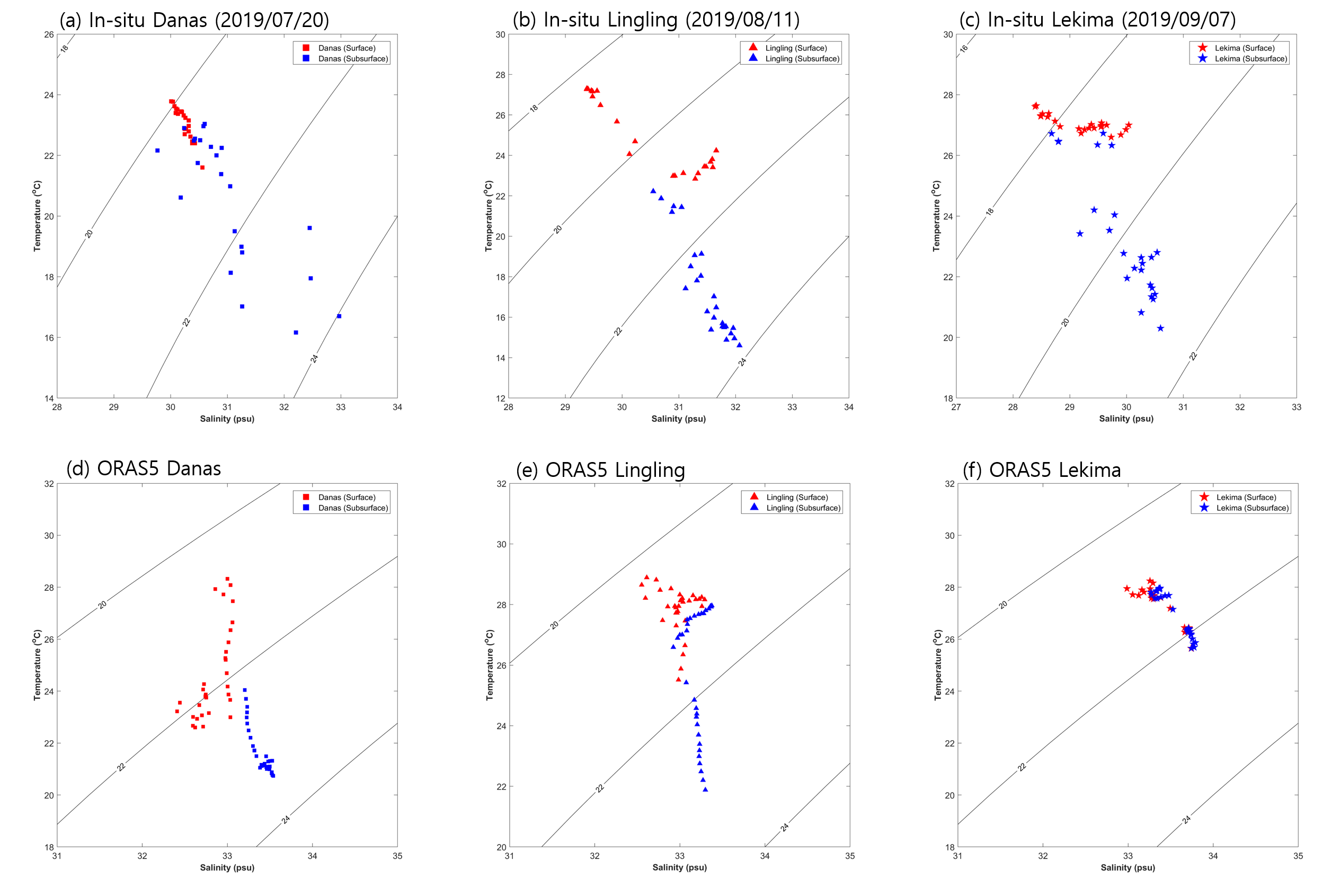


Figure S3. Surface and subsurface T-S diagram observed in IORS for Danas, Lining, and Lekima cases. The in-situ surface observations are measured for hourly interval at 5 m approximately, and the subsurface observations are at 20 m below sea surface for (a) Danas (20 July) and (b) Lingling (11 August). However, in case (c) Lekima (07 September), the subsurface observations are plotted at 40 m due to biofouling at 20 m sensor. In lower panel, the T-S diagram using ORAS5 daily dataset is illustrated from -15 to + 15 days in case (d) Danas, (e) LingLing, and (f) Lekima.


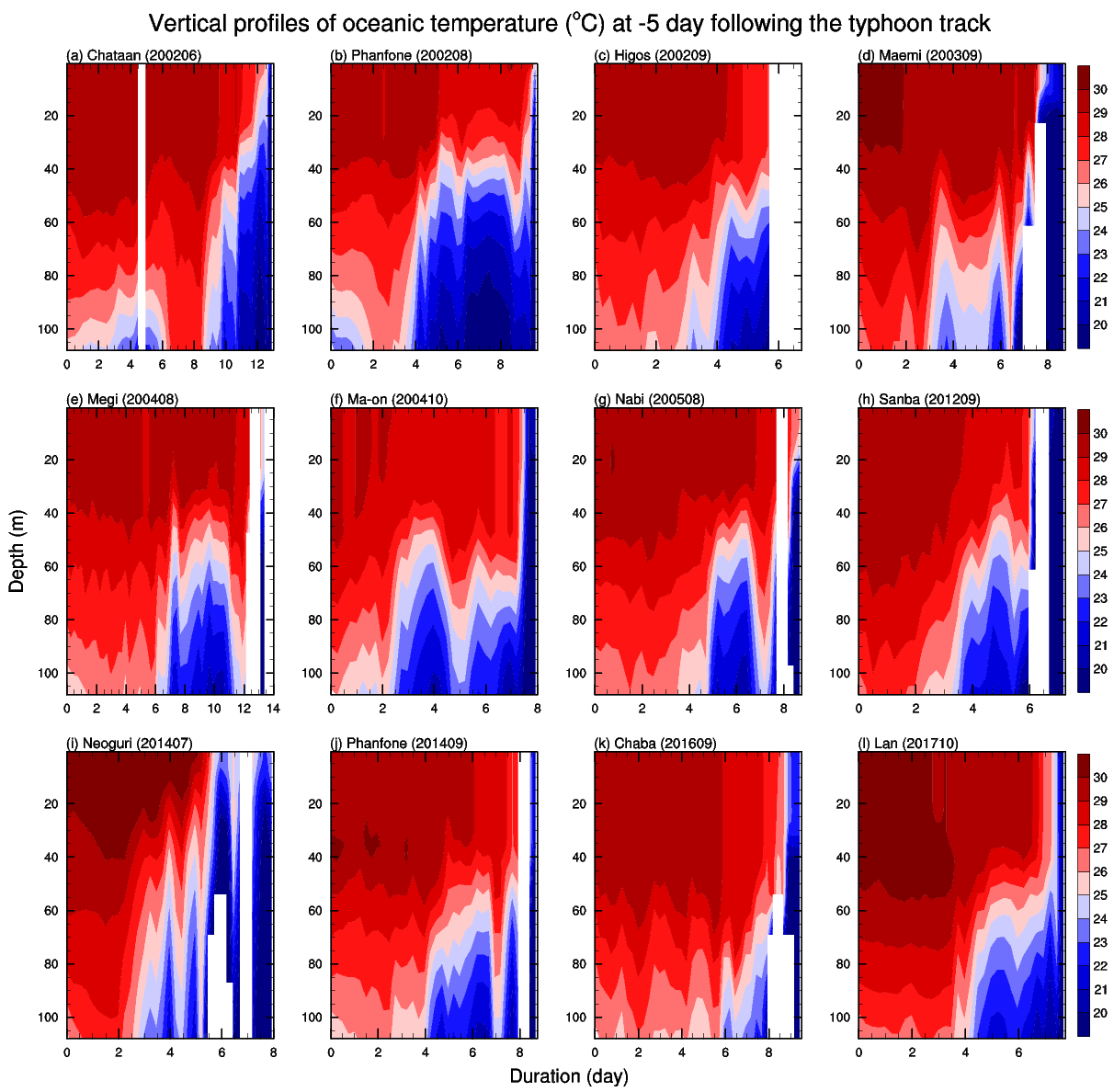


Figure S4. Vertical profiles of ocean temperature following the typhoon track. Shading shows 5 days before each typhoon arrived.


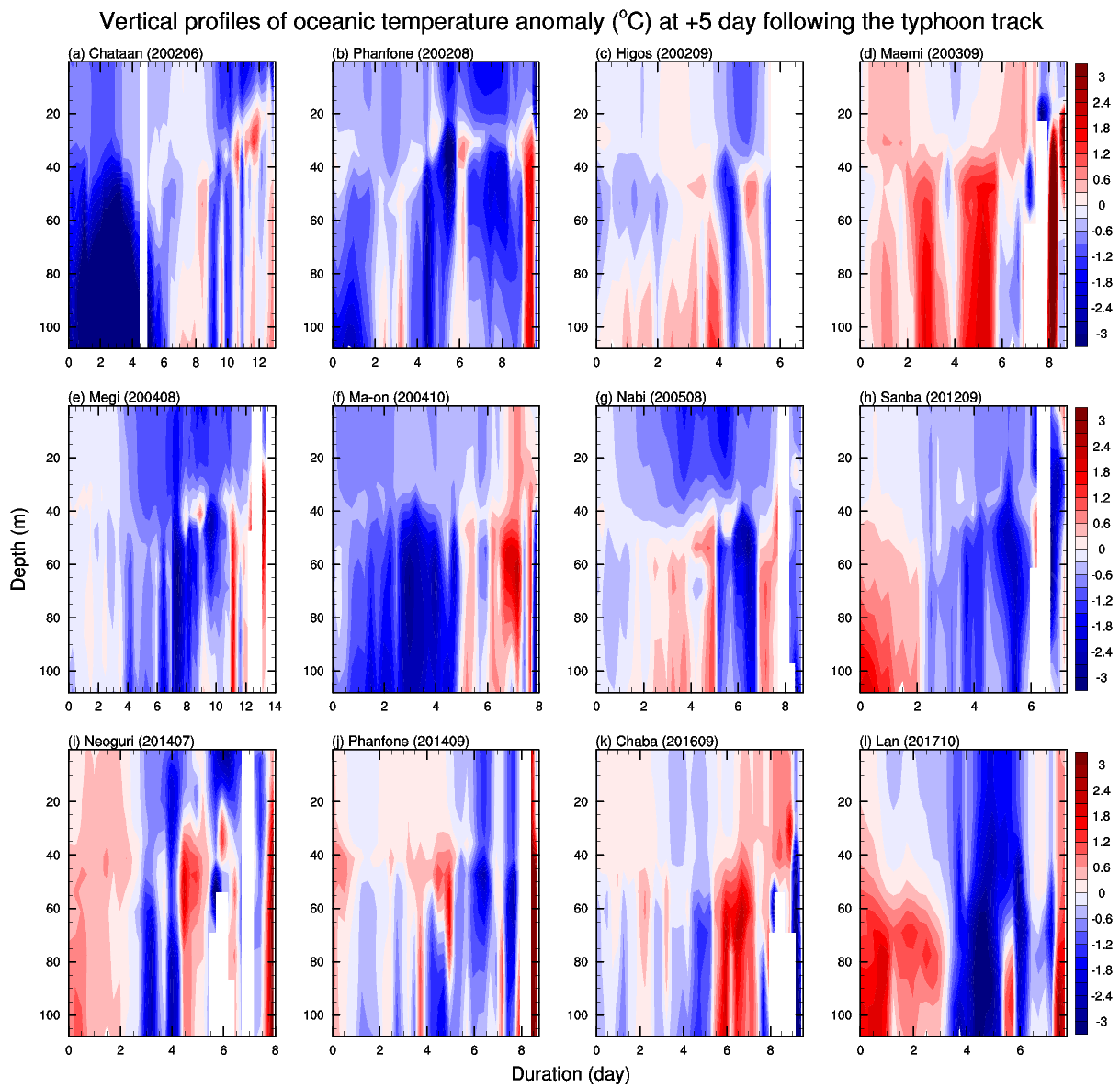


Figure S5. Vertical profiles of oceanic temperature anomaly following the typhoon track. Shading shows 5 days after each typhoon arrived.


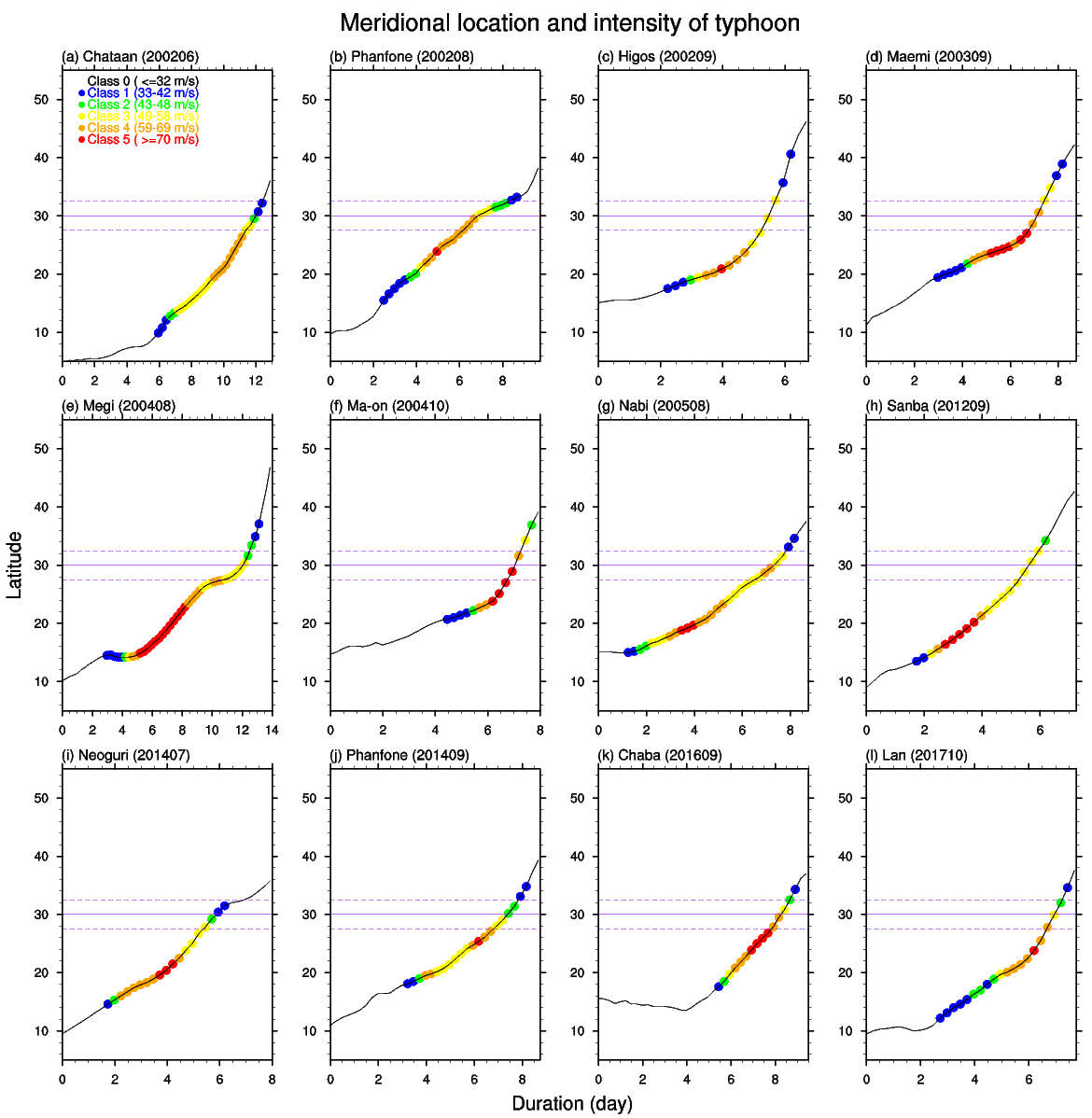


Figure S6. Latitudinal trajectory of typhoons with colors showing the intensity class of typhoons in each time step.


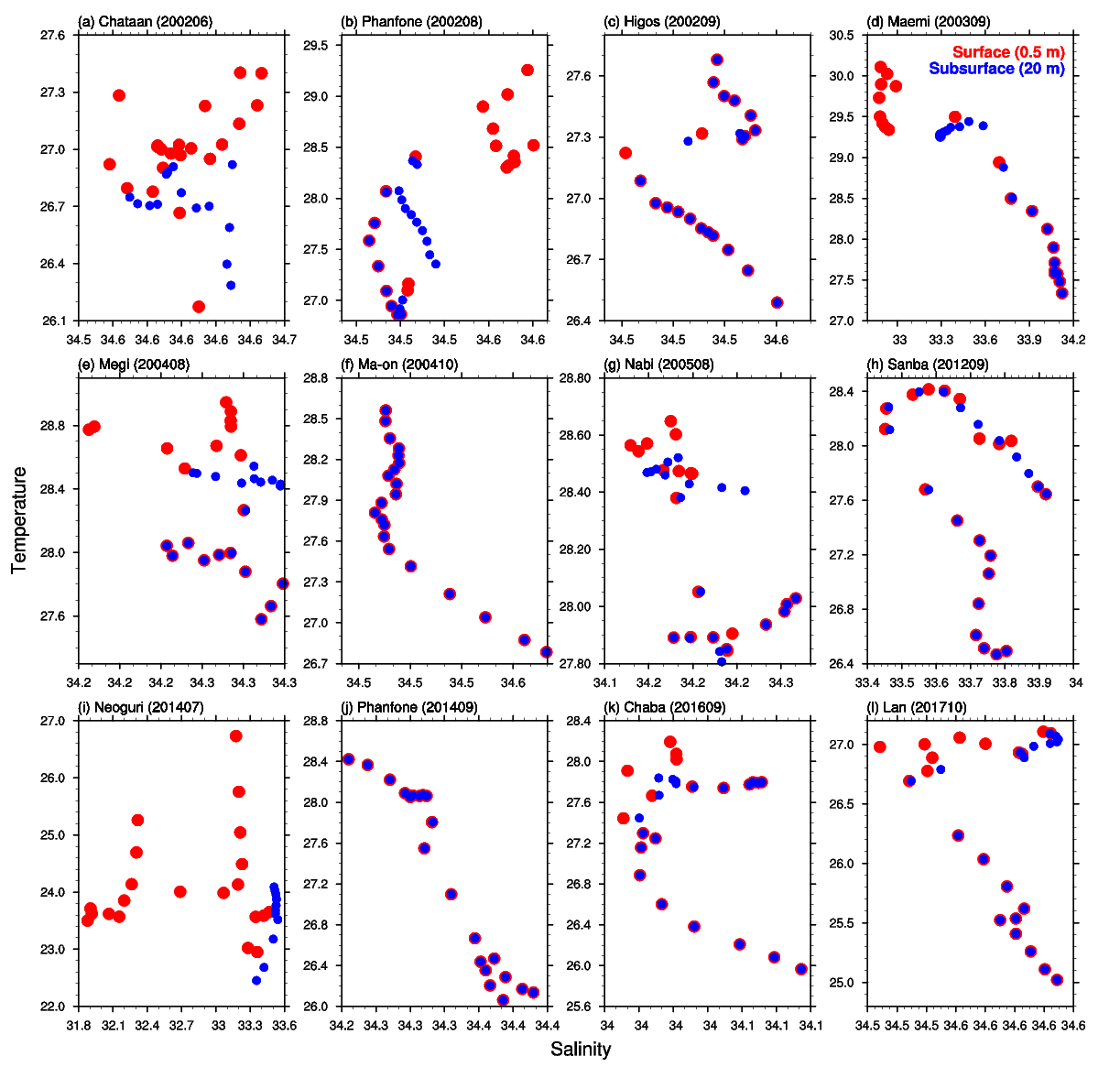


Figure S7. T-S diagram When typhoons are located at 30°N. The surface (red) and subsurface (blue) temperature with salinity are plotted from -10 to 10 days for 12 typhoons.


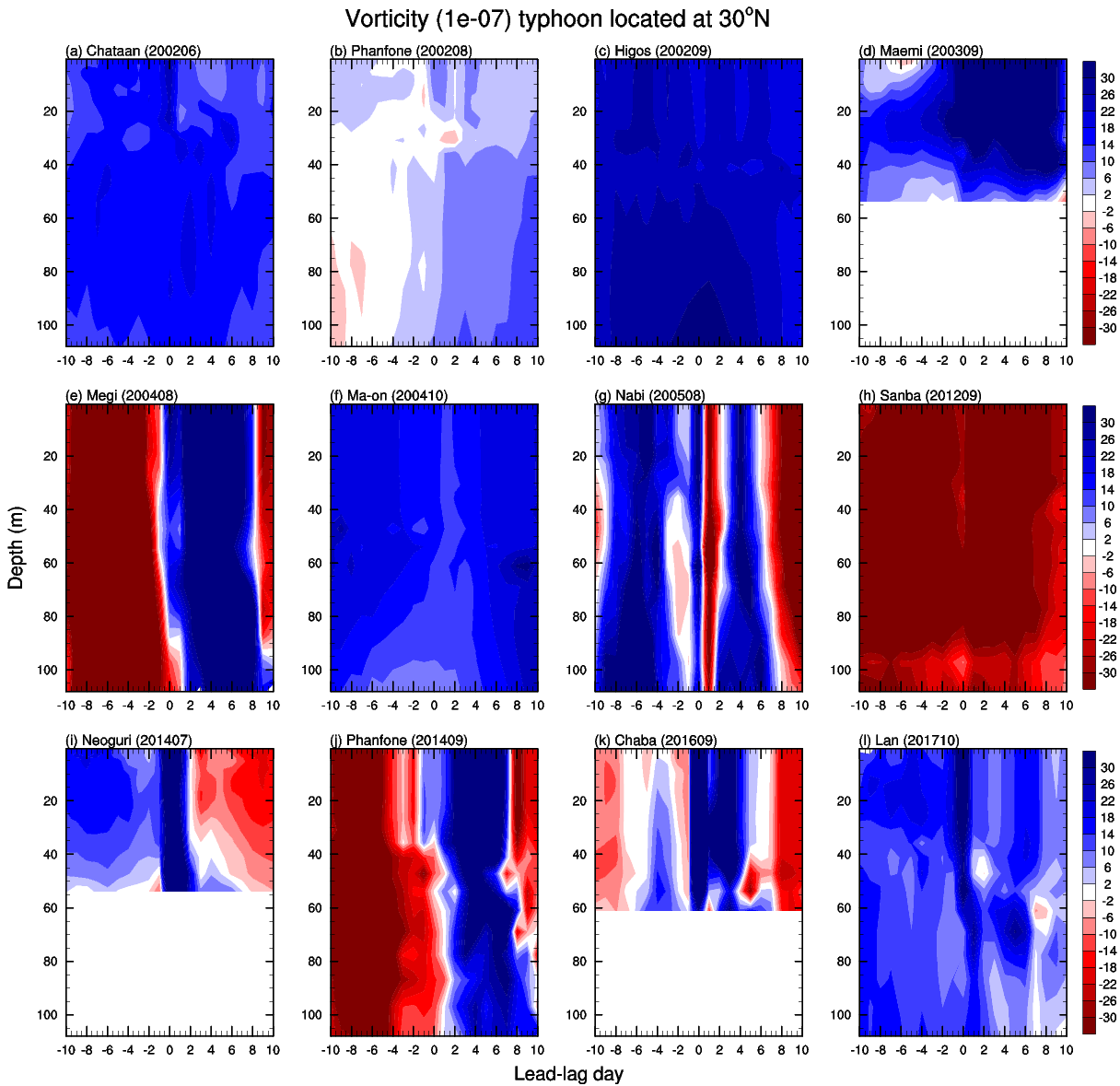


Figure S8. Lead-lag ocean vorticity with respect to typhoon arrival day. When typhoons are located at 30°N, vertical profiles are plotted from -10 to 10 days for 12 typhoons.


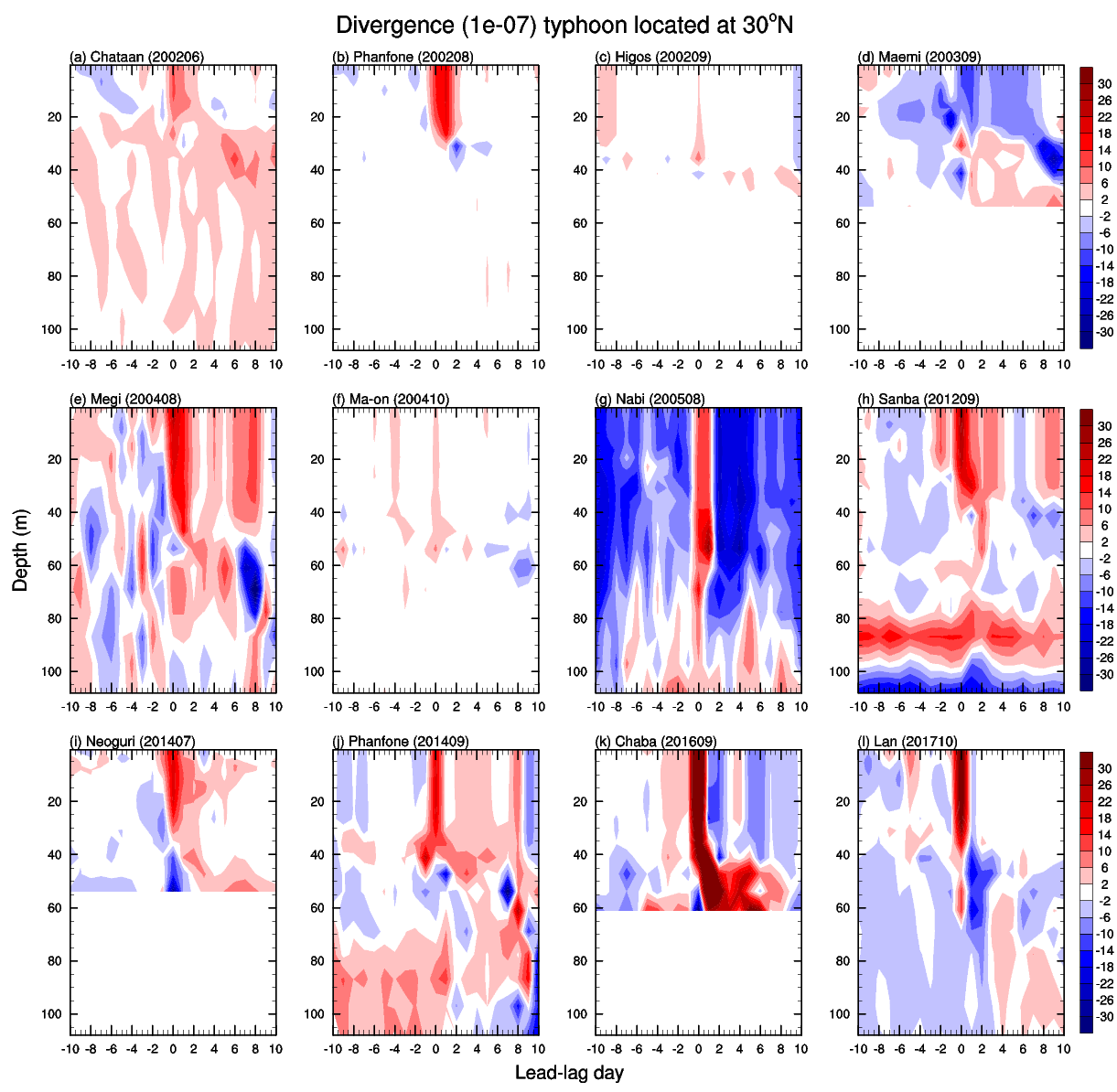


Figure S9. Lead-lag ocean divergence with respect to typhoon arrival day. When typhoons are located at 30°N, vertical profiles are plotted from -10 to 10 days for 12 typhoons.


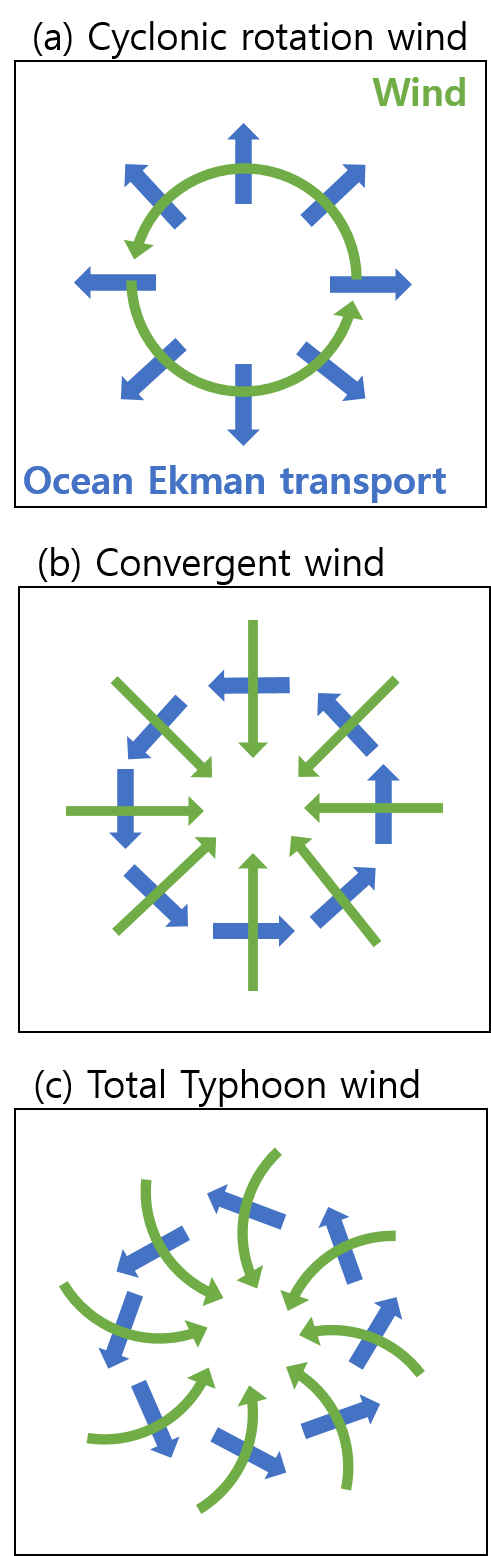


Figure S10. Schematics of the ocean current response to the (a) rotational, (b) convergent, and (c) total typhoon wind forcing. Green and blue arrows denote wind direction and ocean Ekman transport, respectively.
